# 4D deep learning for real-time volumetric optical coherence elastography

**DOI:** 10.1007/s11548-020-02261-5

**Published:** 2020-09-30

**Authors:** M. Neidhardt, M. Bengs, S. Latus, M. Schlüter, T. Saathoff, A. Schlaefer

**Affiliations:** Institute of Medical Technology and Intelligent Systems, Hamburg University of Technology, Hamburg, Germany

**Keywords:** Optical coherence elastography, Deep learning, Convolutional neuronal networks, Real-time imaging

## Abstract

**Purpose:**

Elasticity of soft tissue provides valuable information to physicians during treatment and diagnosis of diseases. A number of approaches have been proposed to estimate tissue stiffness from the shear wave velocity. Optical coherence elastography offers a particularly high spatial and temporal resolution. However, current approaches typically acquire data at different positions sequentially, making it slow and less practical for clinical application.

**Methods:**

We propose a new approach for elastography estimations using a fast imaging device to acquire small image volumes at rates of 831 Hz. The resulting sequence of phase image volumes is fed into a 4D convolutional neural network which handles both spatial and temporal data processing. We evaluate the approach on a set of image data acquired for gelatin phantoms of known elasticity.

**Results:**

Using the neural network, the gelatin concentration of unseen samples was predicted with a mean error of 0.65 ± 0.81 percentage points from 90 subsequent volumes of phase data only. We achieve a data acquisition and data processing time of under 12 ms and 22 ms, respectively.

**Conclusions:**

We demonstrate direct volumetric optical coherence elastography from phase image data. The approach does not rely on particular stimulation or sampling sequences and allows the estimation of elastic tissue properties of up to 40 Hz.

## Introduction

Elasticity of tissue can be used to differentiate between malignant and healthy tissue. Hence, estimating elastic properties of soft tissue can assist physicians in treatment and diagnosis of diseases [1]. Different approaches for elastography have been proposed, including methods measuring the tissue compression and methods estimating the shear wave propagation velocity. The latter is directly related to the shear modulus, resulting in a quantitative value. Given the speed of shear waves in soft tissues, the measurement of the shear wave propagation velocity needs to be sufficiently fast to fulfill the sampling theorem.

A number of image modalities have been studied for shear wave imaging, including magnet resonance imaging [2], ultrasound [1] and optical coherence tomography (OCT). The respective data acquisition schemes depend on the temporal sampling rates and the field of view (FOV). Optical coherence elastography (OCE) is particularly sensitive for small displacements and allows for rather high spatial and temporal resolution. Different approaches to estimate shear wave velocities with OCE have been proposed. Conventional methods detect the signal peak at two positions [3] which is only feasible when the direction of wave propagation is known. Acquiring OCE data at multiple imaging positions in a reverberant shear wave field has also been demonstrated [4]. However, the authors of this recent publication used a triggered sequential data acquisition with 60 s per 4D dataset and explicit data processing methods.

We propose a novel approach that obtains full volumetric OCE images at a rate of 831 Hz. Instead of conventional data processing to estimate shear wave peaks, we employ a specifically designed 4D convolutional neural network to process the phase of the complex OCE images. Our network is trained to predict the concentration of gelatin in different phantoms, and we demonstrate that fast and accurate estimation of elastic tissue properties is feasible independent on the measuring position relative to the excitation point. Also, our novel 4D neural networks are designed to identify temporal patterns without any explicit physical model of the wave propagation or assumptions about the wave type. Our approach could potentially differentiate precisely between tumor and surrounding tissue in real time during minimal invasive surgery.

**Fig. 1:**
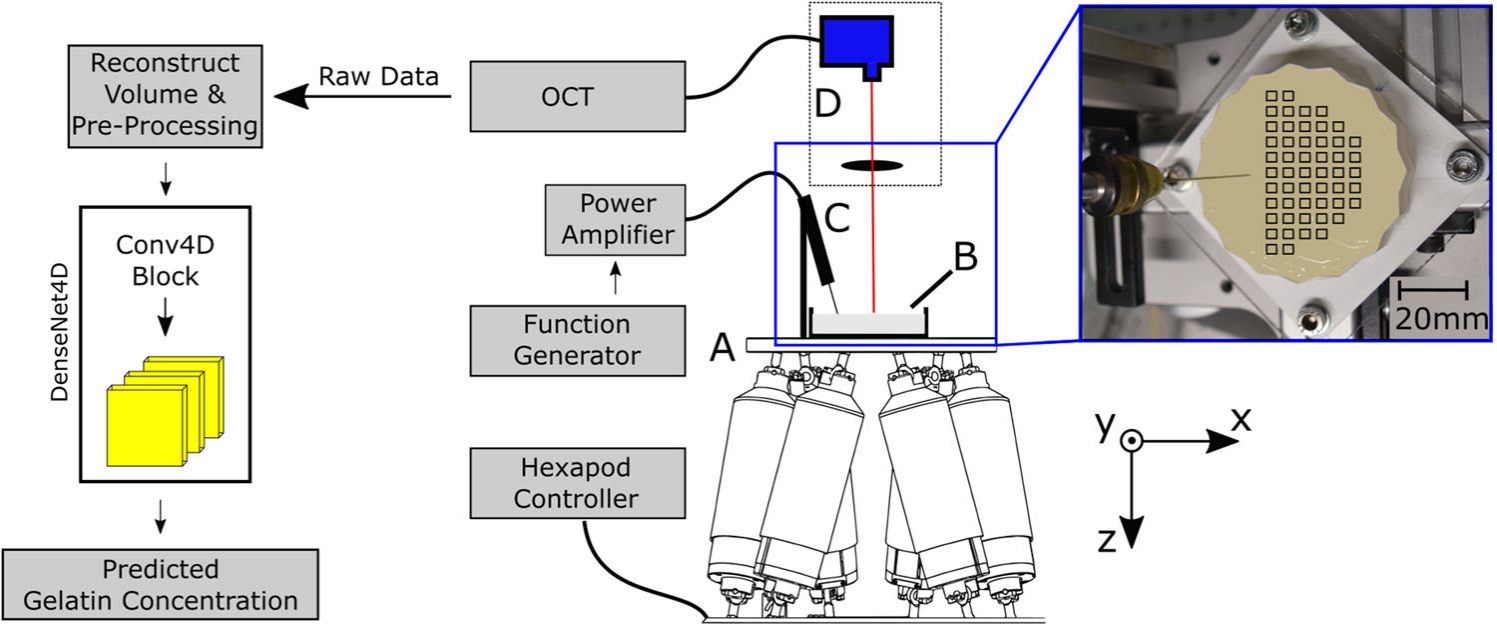
Experimental setup: a robot **a** drives a scanning profile along the *x*- and *y*-axes. Shear waves are excited continuously inside a gelatin phantom **b** through a needle connected to a piezoelectric actuator (**c**). An OCT scan head (**d**) acquires volumes with a frequency of 831 Hz at the positions indicated by the black rectangles on the image (right). Raw data are reconstructed and pre-processed, and gelatin concentration is estimated with a 4D deep learning network

**Fig. 2:**
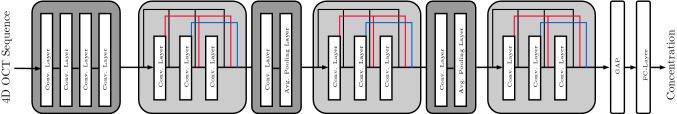
Our architecture predicts gelatin concentration in an end-to-end fashion using a 4D OCT sequence. The architecture consists of an initial part with four convolutional layers, followed by three DenseNet blocks, which are connected with transition layers. The last block is connected to a global average pooling (GAP) layer, and the output is fed into the regression output layer

**Table 1:** Results for predicting gelatin concentration

Num. of sequences	MAE (p.p.)	rMAE (p.p.)	PCC	Inference time (ms)
1 (10 volumes)	0.715 ± 0.938	0.143 ± 0.188	0.973	21.65 ± 0.19
3 (30 volumes)	0.675 ± 0.819	0.135 ± 0.164	0.978	55.76 ± 0.15
9 (90 volumes)	0.655 ± 0.812	0.131 ± 0.162	0.980	148.6 ± 0.05

Each sequence consists of ten subsequent volumes which we input into our network. We evaluate a varying number of sequences. Prediction results are average when multiple sequences are used

**Table 2:** Mean and standard deviation in estimating gelatin concentration with a sequence length of ten subsequent volumes

Gelatin concentration	5%	7.5%	10%	12.5%	15%	17.5%	20%
Mean estimation [%]	5.47	7.59	9.80	12.77	13.60	17.25	19.67
Mean estimation error [p.p.]	0.55	0.31	0.89	0.65	1.40	0.52	0.67
SD	0.93	0.70	0.95	0.87	0.81	0.66	1.19

## Methods

*Dataset* We employ a high-speed OCE imaging system (OMES, OptoRes, Germany) with a scan rate of 1.59 MHz and define a scan line as a one-dimensional depth resolved signal. An optical scanner deflects scan lines along the *x*- and *y*-axes resulting in a volume size of $3\times 3\times 2$ mm in air ($32 \times 32 \times 470$ pixels) along the *x*-, *y*- and *z*-axes, respectively. A continuous shear wave field is induced with a needle (gauge 21) attached to a piezoelectric actuator ($f=$ 100 Hz). Phantom and actuator are mounted onto a robot which allows us to position the FOV on the phantom. The position of the needle is not changed in the phantom as we move the FOV relative to the needle. The overall experimental setup is depicted in Fig. 1. Note, the advantage of our approach is that no synchronization is required since wave propagation is covered in the sequence of volumetric images.

For data acquisition, we record at each position 90 subsequent volumes with a temporal rate of 831 Hz, which we define as a 4D OCE data sequence. For each gelatin to water concentration (5.00%, 7.50%, 10.00%, 12.5%, 15.00%, 17.5% and 20.00%), we produce six phantoms. We established the gelatin elasticity using mechanical indentation tests similar to [5]. The elasticities of our phantoms range from 21 to 119 kPa which is similar to the elasticity of benign and malignant prostate tissue (24–92 kPa) [6]. Note that we report concentrations, as they correspond to simpler values used when creating the phantoms. A 4D OCE data sequence is acquired at the indicated 52 positions in Fig. 1 for each phantom.

The 4D OCE data sequence is pre-processed by detecting the surface of the phantom as an intensity peak. Next, the phase part is extracted since this data type includes information on the wave travelling through our FOV. We crop volumes along the depth axis (*z*-axis) to 250 px beneath the surface and unwrap the phase between subsequent volumes. Last, each volume is resized to $32 \times 32 \times 32$ pixels along the *x*-, *y*- and *z*-axes, respectively, to reduce computation time and memory requirements. Note that we do not apply any specific calculations for data pre-processing except for phase unwrapping and only use phase data for training our networks.

*Deep learning methods* To estimate gelatin concentration based on 4D OCE data in an end-to-end fashion, we use a 4D spatiotemporal convolutional neural network, which jointly learns from the spatial and temporal dimensions by using 4D convolutions as the network operations. As a baseline, we consider a densely connected neural network (DenseNet) [7], due to its parameter and computational efficiency, which is particularly relevant for the challenging problem of 4D deep learning. Also, a similar architecture has been used for gelatin concentration prediction based on 3D OCE data [8]. Next, we refine the architectures components, using our validation dataset. We use an initial convolutional part with four convolutional layers, followed by our DenseNet architecture, which consists of three DenseNet blocks with a feature growth rate of 8. Each of the DenseNet blocks consists of three convolutional layers, while each layer is connected to all its proceeding layers within one block. To preserve the input size throughout the convolutional layers, we use zero padding of the inputs. For connecting the DenseNet blocks and for downsampling of our input dimensions, we use average pooling layers with a stride of 2. Also, we use batch normalization [9] for all our convolutional layers and employ the rectified linear activation function for our network layers. After the last DenseNet block, we employ a global average pooling layer and connect the linear regression output layer for predicting the gelatin concentration. Our final architecture is shown in Fig. 2. Note that we used TensorFlow for our implementation.

For our deep learning approach, we consider sequences of ten subsequent volumes cropped from 4D OCE sequences with a length of 90. During training, we loop through our training data and randomly crop subsequences with a length of 10 from the entire OCE sequences. In this way, we are able to augment our training dataset size by using random temporal cropping during training. We do not apply any additional data augmentation, such as rotations of the volumes. We train our network for 1000 iterations with a batch size of 13, using Adam for optimization combined with a mean squared error (MSE) loss function between our predictions and the target labels. For evaluation, we use ordered temporal crops and average the results to obtain one final prediction for an entire sequence. We randomly split our data to avoid overfitting. We use data from four different phantoms from each concentration for training and data from two independent phantoms from each concentration for test and validation, respectively.

**Fig. 3:**
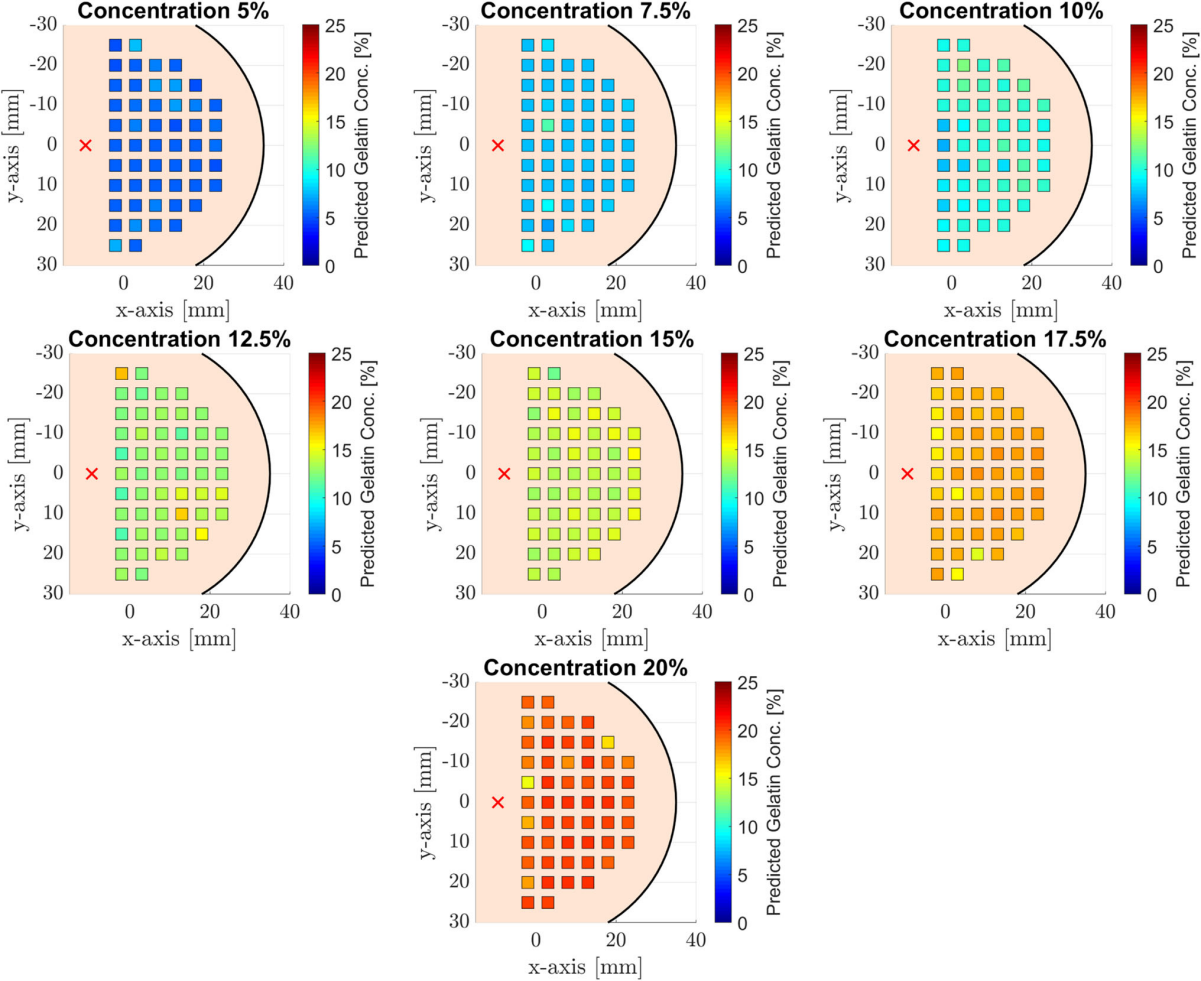
Heat maps show distribution of gelatin concentration estimations. The red ‘*x*’ indicates the excitation position of the shear waves

## Results

Mean and standard deviation for the predicted gelatin concentration are given in Table 1. Our results demonstrate that performance is improved when more sequences are used. On average, the gelatin concentration can be estimated with a mean absolute error (MAE) of 0.66 ± 0.81 percentage points and a Pearson correlation coefficient (PCC) of 98% with a total input sequence length of 90 volumes. The relative mean absolute error (rMAE) is 0.131 ± 0.162 percentage points. Note that the rMAE is relative to the target’s standard deviation.

The inference time ranges from 22 to 149 ms for sequences of length 10, 30 and 90 volumes. Mean and standard deviation for the predicted gelatin concentration with an input sequence of 10 volumes are given in Table 2. Figure 3 shows the spatial distribution of the estimated concentrations. Each phantom has the same scale in percent concentration, and the colored squares represent the measured volumes, and the red crosshairs denote the position of the excitation. While concentrations 10% and 17.5% show a slightly increased error close to the origin of the waves, the remaining concentrations show a slightly increased error at inconsistent positions. This indicates that single estimates are affected by phantom inclusions or inhomogeneities. Hence, no clear dependency between estimate accuracy and the relative position with respect to the origin of the waves can be derived.

## Conclusion

We demonstrate that elastic properties can be estimated from 4D OCE data using deep learning. Our new approach uses fast volumetric imaging of shear wave fields without any assumptions regarding the spatial wave propagation and no need for temporal triggering and binning. Considering a data acquisition time of approximately 12 ms and an inference time of approximately 22 ms, elastography can be realized with up to 40 Hz and small delays. Hence, the proposed setup would be particularly interesting for clinical applications outside controlled laboratory environments.
